# Supplementary material for: Pollution‐Driven Selection in a Non‐Biting Midge: Genome‐Wide Responses to Bacillus thuringiensis israelensis and Copper
Source: Mol Ecol. 2026 Feb 6;35(3):e70263. doi: 10.1111/mec.70263 (PMC12881711; doi:10.1111/mec.70263)
Supplement: Supplementary file 1 — Data S1: mec70263‐sup‐0001‐FigureS1‐S9‐TableS1‐S4.pdf. [file MEC-35-e70263-s001.pdf]

MOLECULAR ECOLOGY

Supplemental Information for:

Pollution-Driven Selection in a Non-biting Midge: Genome-Wide Responses to *Bacillus thuringiensis israelensis* and Copper

Nina Röder, Sara Kolbenschlag, Sebastian Pietz, Reid S. Brennan, Mirco Bundschuh, Markus Pfenninger, Klaus Schwenk

Table of Contents:

**Figure S1** Results from preliminary tests: Bti concentrations	Page 2
**Figure S2** Results from preliminary tests: copper concentrations	Page 3
**Figure S3** Results from preliminary tests: Bti toxicity degradation	Page 4
**Figure S4** Results from preliminary tests: sediment copper concentrations	Page 5
**Figure S5** Number of egg masses sampled per treatment	Page 6
**Figure S6** Candidate SNPs under stricter significance thresholds	Page 6
**Figure S7** Shared response and selection components across replicate combinations	Page 7
**Figure S8** Genome-wide distribution of shared covariance across genomic windows	Page 8
**Figure S9** Relationship between genomic scores and female protein content	Page 9
**Table S1** Summary statistics of variance partitioning results	Page 10
**Table S2** Results of Gene Ontology (GO) enrichment analysis	Page 15
**Table S3** Pearson correlation between genomic scores and phenotypic endpoints: Bti	Page 17
**Table S4** Pearson correlation between genomic scores and phenotypic endpoints: copper	Page 18

MOLECULAR ECOLOGY

Figure S1

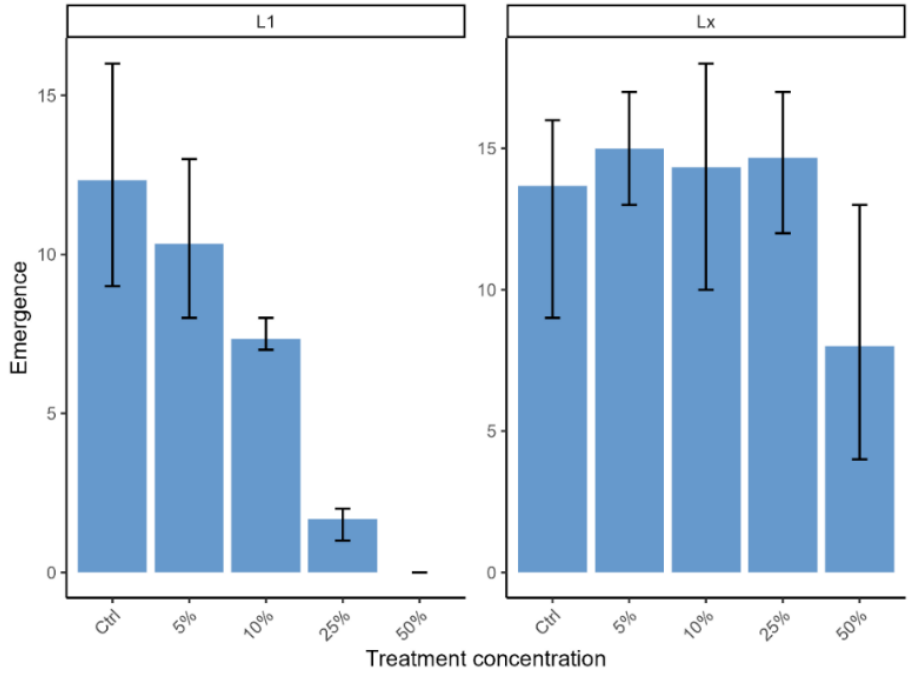

Figure S1 Results from preliminary tests showing the number of emerged chironomid individuals per replicate (mean ± min/max, n = 3) following Bti exposure at different concentrations. 'L1' represents first-instar larvae (1–2 days post-hatching), and 'Lx' represents later-stage larvae (7–8 days post-hatching) at Bti application. An intermediate concentration of 33% was chosen to avoid complete mortality of L1 larvae while still aiming to induce effects in Lx larvae.

MOLECULAR ECOLOGY

Figure S2

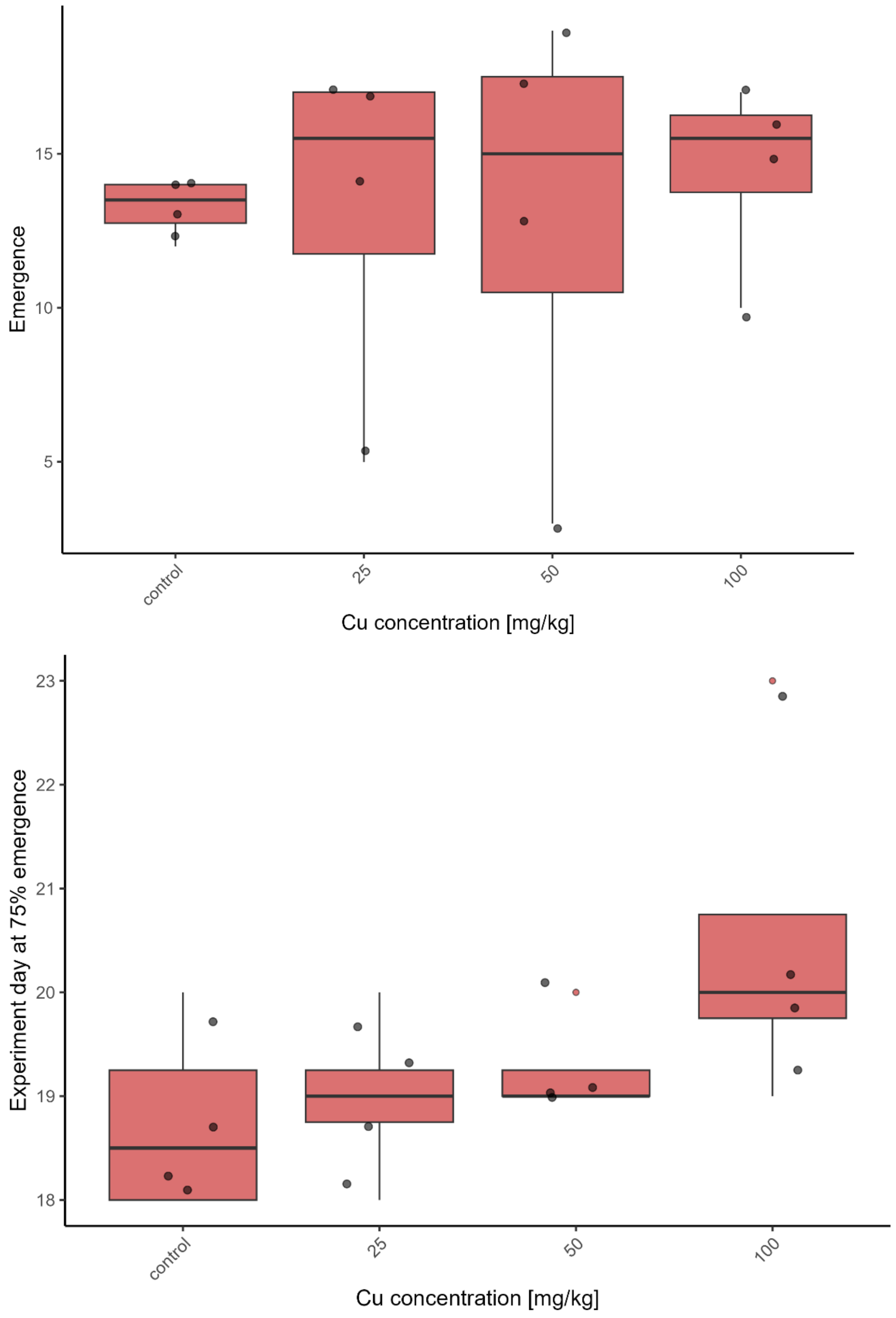

Figure S2 Results from preliminary tests showing the effects of different nominal copper concentrations on chironomid emergence. Top panel: Number of emerged chironomid individuals per replicate (mean ± SD, n = 4). Bottom panel: Experiment day at which 75% of individuals had emerged (mean ± SD). A concentration of 100 mg/kg Cu was selected, as delayed emergence at this concentration indicated sublethal effects, while total emergence was similar across all treatments. Delayed emergence was particularly pronounced in females, whereas the male-to-female ratio remained unaffected by treatment.

MOLECULAR ECOLOGY

Figure S3

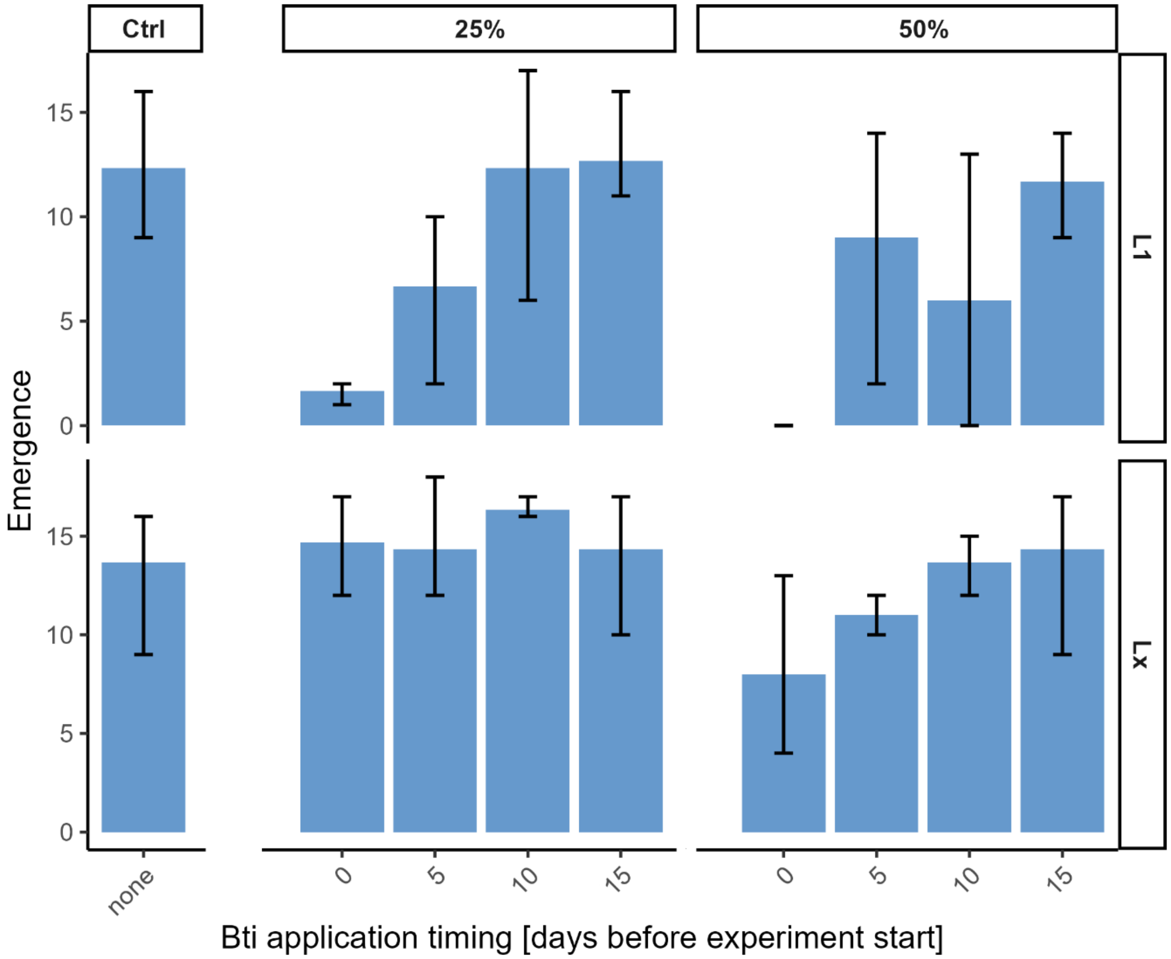

Figure S3 Results from preliminary tests showing the effect of the time interval between Bti application and chironomid exposure, as a measure of toxicity degradation. L1 larvae were first-instar larvae (1–2 days post-hatching), and Lx larvae were later-stage larvae (7–8 days post-hatching). Chironomids were exposed to Bti at different concentrations (25% and 50%), and emergence was measured for each replicate (mean ± min/max, n = 3). No effect on emergence was observed when larvae were introduced to the experimental vessels 10 or 15 days after Bti application.

MOLECULAR ECOLOGY

Figure S4

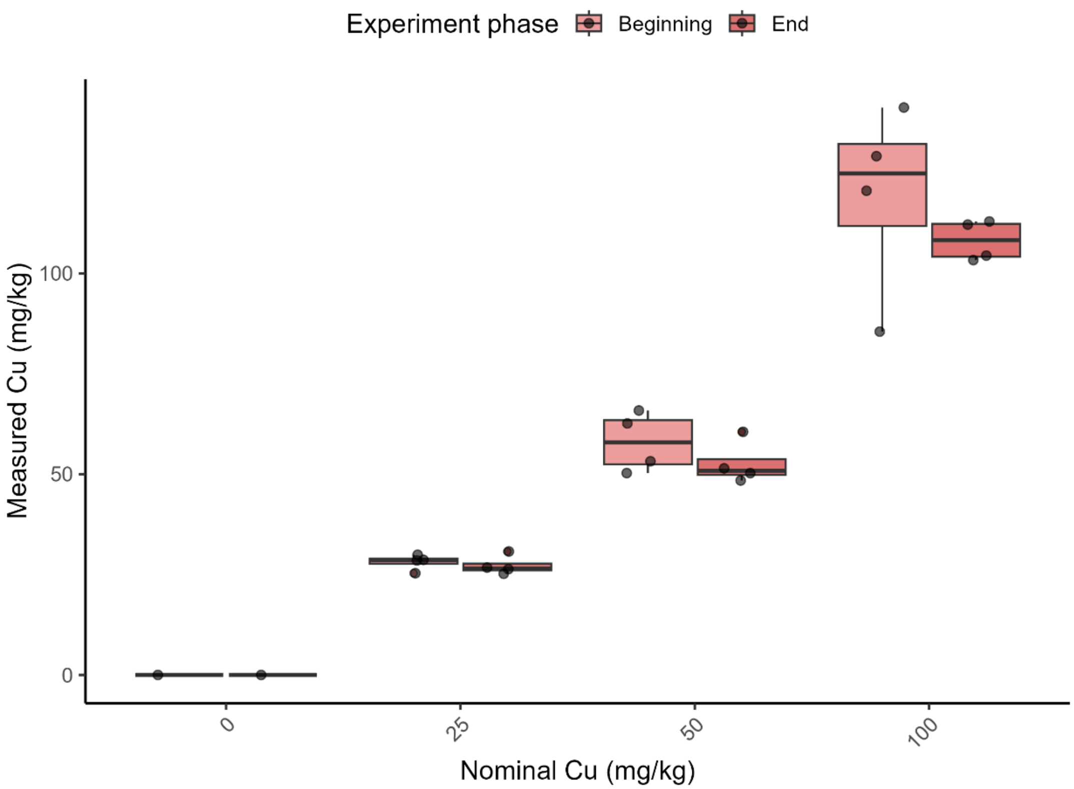

Figure S4 Results from preliminary tests showing the measured Cu concentrations in sediment for different nominal Cu treatments (mean ± SD, n = 4) at the beginning of the pilot experiment and after 40 days of exposure with chironomid larvae. Replicates experienced medium exchanges every 10–14 days, with half receiving fresh medium spiked with Cu in the water phase based on copper measurements of the replaced medium. Only the nominal Cu concentration affected measured sediment concentrations, independent of medium exchange, Cu spikes in the water, or sampling time point.

MOLECULAR ECOLOGY

Figure S5

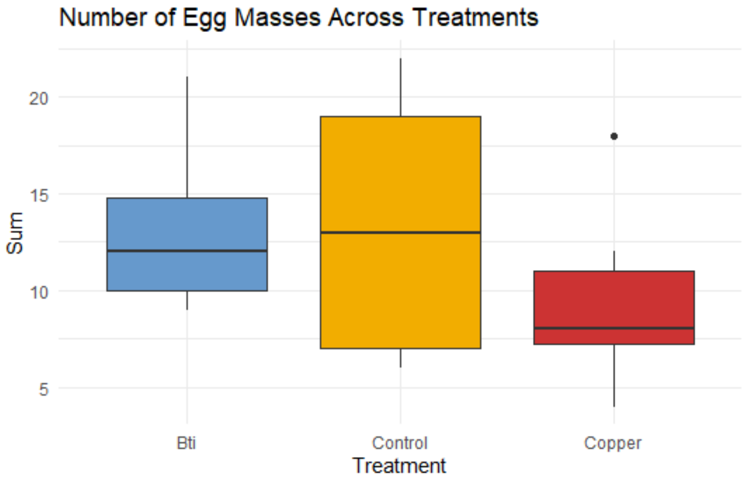

Figure S5 Number of egg masses sampled per treatment (n = 6) following 26 weeks of chronic exposure. Egg masses were collected over three consecutive days and stored in SAM-5S medium until hatching. Larvae hatching from these egg masses were used for population genomic analyses. Boxplots indicate the interquartile range (IQR), horizontal lines denote the median, whiskers extend to 1.5× IQR, and points beyond this range are shown as outliers.

Figure S6

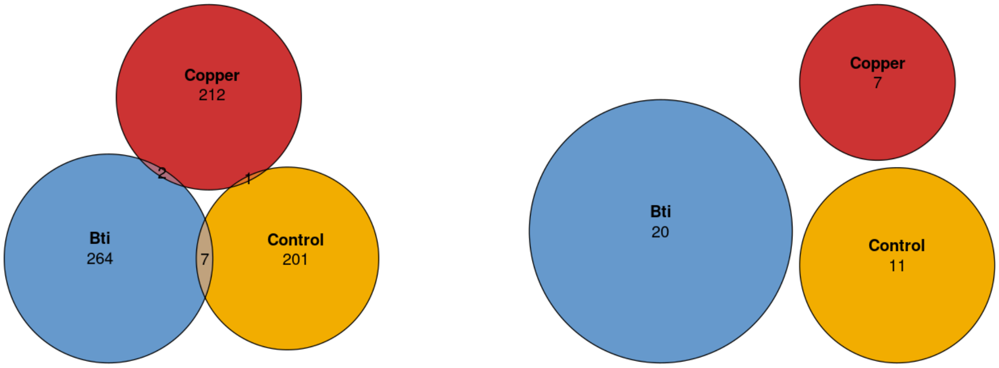

Figure S6 Venn diagrams showing the number of candidate SNPs under stricter significance thresholds: (left) ≥5 of 6 replicates, and (right) all 6 replicates per treatment. Stricter criteria greatly reduced both treatment-specific and shared SNPs.

MOLECULAR ECOLOGY

Figure S7

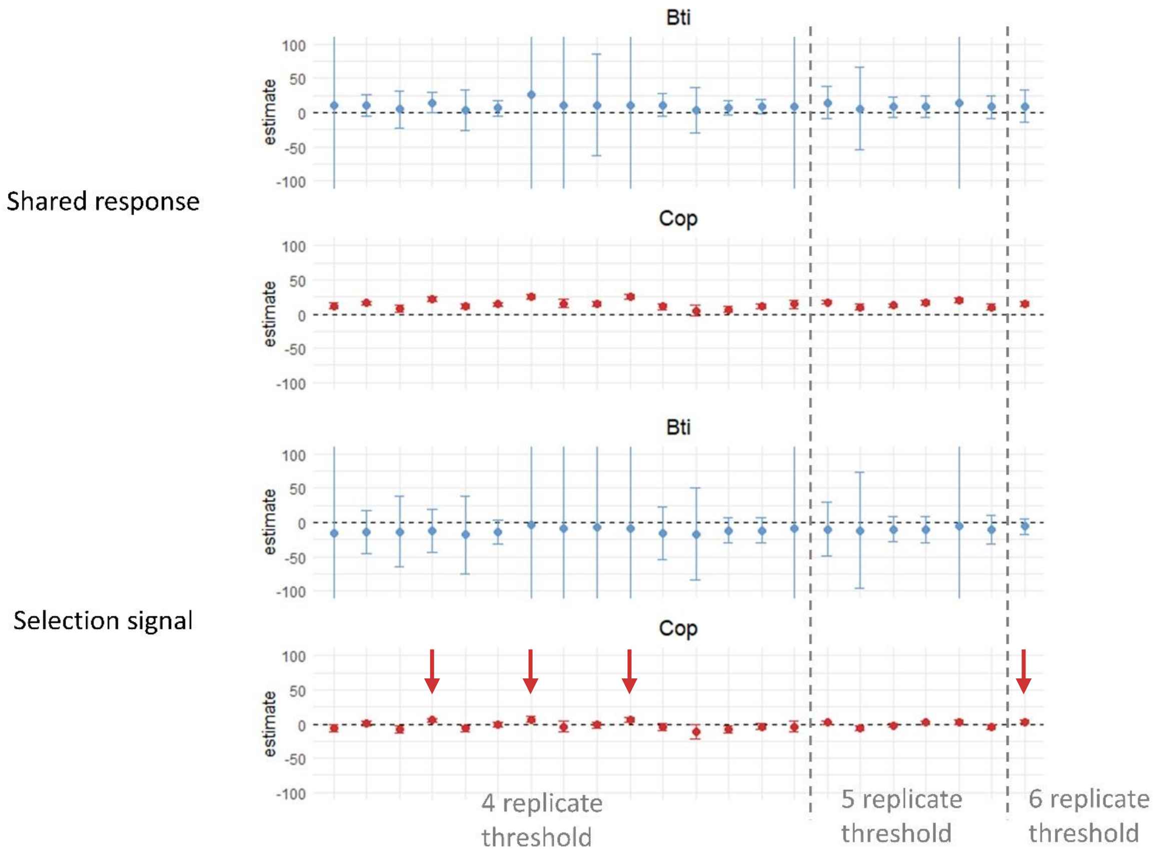

Figure S7 Partitioning of allele frequency change variance into shared and selection components across replicate combinations for Bti- and copper-treated populations. Upper panels show the percentage of total variance attributed to shared allele frequency change (i.e., parallel change), and lower panels show the variance attributed to selection after accounting for laboratory adaptation. Statistically significant positive selection estimates are highlighted with arrows. Each point represents a replicate combination (15 four-replicate, 6 five-replicate, and 1 six-replicate combination per treatment), with 95% confidence intervals indicated by error bars. Variance components are expressed as percentages of total variance in allele frequency change from F0 to F8. Error bars extending beyond the plotting range (±100%) are truncated visually.

MOLECULAR ECOLOGY

Figure S8

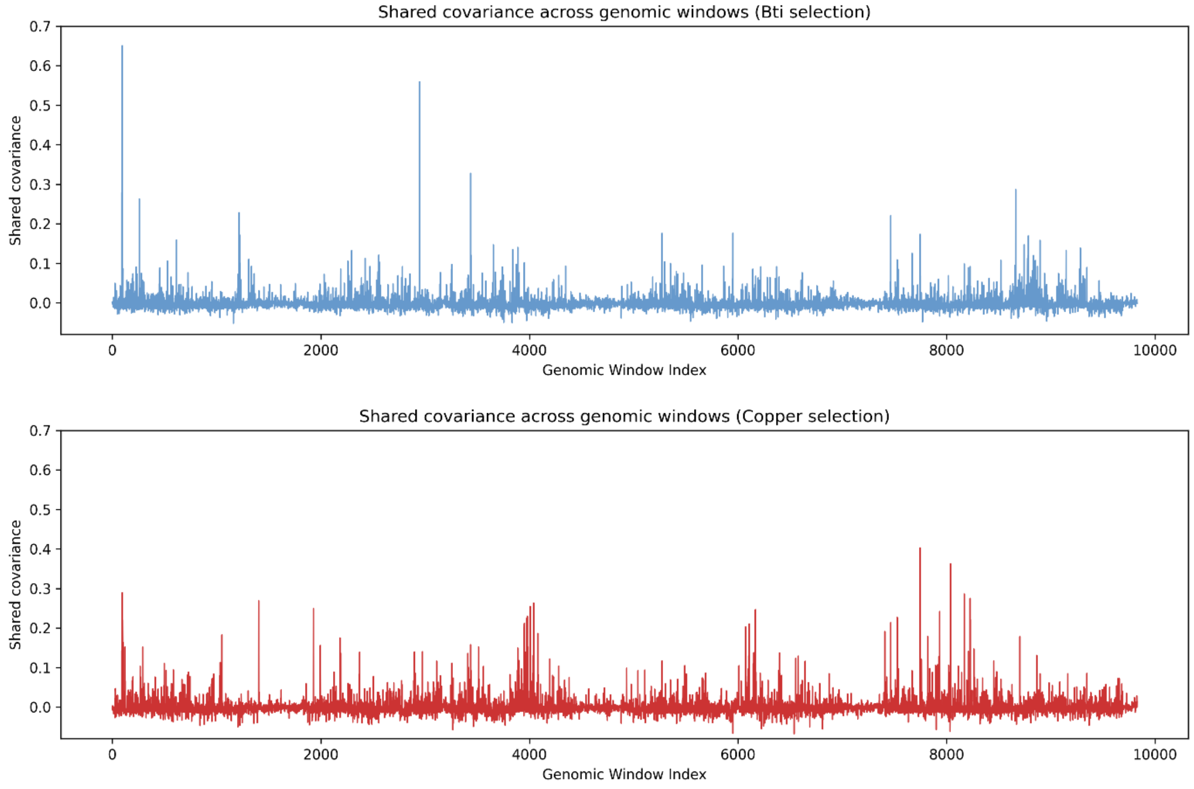

Figure S8 Genome-wide distribution of shared covariance across genomic windows in six-out-of-six Chironomus riparius *populations exposed to Bti (top panel) and copper (bottom panel). For each genomic window, the proportion of total genetic variance attributable to shared allele frequency changes across replicates is shown.*

MOLECULAR ECOLOGY

Figure S9

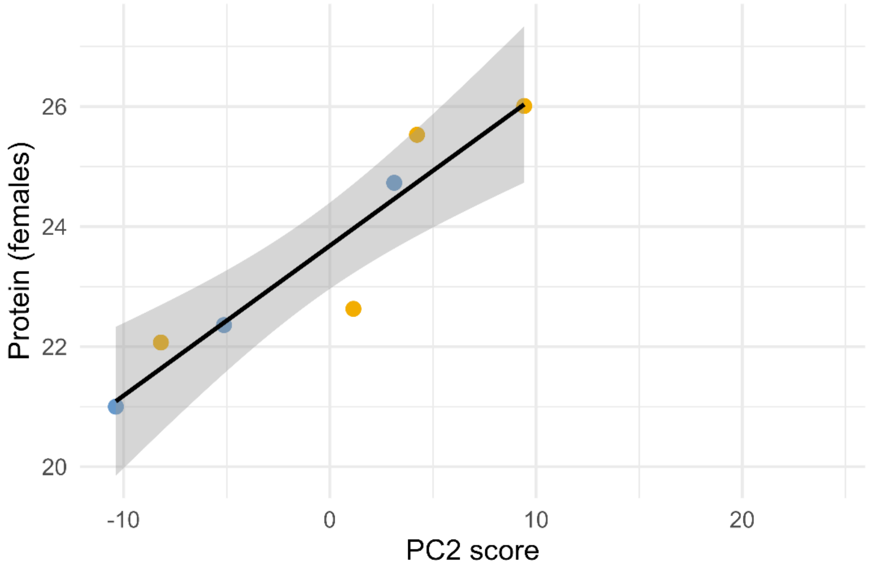

Figure S9 Relationship between PC2 scores and female protein content in C. riparius adults, when exposed to 33% FR Bti during a later larval stage on day 10 of the sensitivity test. Each point represents a replicate population, colored by exposure history (yellow: naive and blue: pre-exposed). The black line shows the fitted linear regression with 95% confidence intervals (shaded area). PC2 scores represent variation in genome-wide allele frequencies identified by principal component analysis (PCA). Protein content (% dry weight) was measured in two technical replicates of one to two adult females emerging from each replicate population (for details see Kolbenschlag et al., 2024). As protein content and PC2 scores appear independent of the exposure history, this pattern likely reflects lab adaptation rather than stressor-specific selection.

MOLECULAR ECOLOGY

Table S1

Table S1 Summary statistics of variance partitioning results for all tested replicate combinations across treatments. Each row reports the estimate and confidence interval (lower and upper error bounds) for one of four variance components—total variance in allele frequency change, the shared response, the portion attributable to laboratory adaptation, and the inferred contribution of experimental selection—for a specific replicate combination in either Bti-treated or copper-exposed populations. The combination number refers to a specific set of replicates, and the number of replicates used in each is indicated. The range column reflects the span between the upper and lower error bounds. See Supplementary Figure S2 for graphical visualization of the shared and selection variance components.

Sort	treatment	variable	combination number	lower_err	estimate	upper_err	range	based on .. replicates
1	Bti	total	1	0.0169	0.0178	0.0188	0.0019	4
2	Bti	shared	1	-618.215	10.951	640.118	1258.333	4
3	Bti	lab	1	-1147.781	26.757	1201.296	2349.077	4
4	Bti	selection	1	-1818.411	-15.806	1786.799	3605.21	4
5	Bti	total	2	0.0198	0.0209	0.022	0.0022	4
6	Bti	shared	2	-4.19	11.295	26.78	30.97	4
7	Bti	lab	2	4.008	24.414	44.82	40.812	4
8	Bti	selection	2	-44.733	-13.119	18.496	63.229	4
9	Bti	total	3	0.0196	0.0206	0.0216	0.002	4
10	Bti	shared	3	-21.682	4.925	31.531	53.213	4
11	Bti	lab	3	-10.192	18.075	46.341	56.533	4
12	Bti	selection	3	-64.265	-13.15	37.965	102.23	4
13	Bti	total	4	0.0196	0.0207	0.0217	0.0021	4
14	Bti	shared	4	0.519	15.037	29.555	29.036	4
15	Bti	lab	4	4.995	27.112	49.229	44.234	4
16	Bti	selection	4	-44.032	-12.074	19.883	63.915	4
17	Bti	total	5	0.0194	0.0204	0.0214	0.002	4
18	Bti	shared	5	-26.713	3.348	33.409	60.122	4
19	Bti	lab	5	-9.36	20.738	50.836	60.196	4
20	Bti	selection	5	-73.975	-17.39	39.195	113.17	4
21	Bti	total	6	0.0224	0.0235	0.0246	0.0022	4
22	Bti	shared	6	-5.543	6.489	18.521	24.064	4
23	Bti	lab	6	9.459	19.443	29.426	19.967	4
24	Bti	selection	6	-30.425	-12.953	4.518	34.943	4
25	Bti	total	7	0.014	0.015	0.0159	0.0019	4
26	Bti	shared	7	-6455.978	26.603	6509.184	12965.162	4
27	Bti	lab	7	-206971.779	29.687	207031.152	414002.931	4
28	Bti	selection	7	-203467.4	-3.084	203461.232	406928.632	4
29	Bti	total	8	0.0138	0.0147	0.0155	0.0017	4
30	Bti	shared	8	-4093.184	10.619	4114.422	8207.606	4

31	Bti	lab	8	-12646.692	18.289	12683.27	25329.962	4
32	Bti	selection	8	-16774.891	-7.671	16759.55	33534.441	4
33	Bti	total	9	0.0168	0.0178	0.0187	0.0019	4
34	Bti	shared	9	-63.186	11.572	86.33	149.516	4
35	Bti	lab	9	-163.396	18.1	199.597	362.993	4
36	Bti	selection	9	-258.171	-6.528	245.115	503.286	4
37	Bti	total	10	0.0166	0.0175	0.0185	0.0019	4
38	Bti	shared	10	-150.599	10.841	172.281	322.88	4
39	Bti	lab	10	-283.03	19.377	321.785	604.815	4
40	Bti	selection	10	-469.503	-8.537	452.43	921.933	4
41	Bti	total	11	0.019	0.02	0.021	0.002	4
42	Bti	shared	11	-4.597	11.452	27.5	32.097	4
43	Bti	lab	11	0.442	26.251	52.059	51.617	4
44	Bti	selection	11	-53.086	-14.799	23.488	76.574	4
45	Bti	total	12	0.0187	0.0197	0.0207	0.002	4
46	Bti	shared	12	-29.799	3.26	36.32	66.119	4
47	Bti	lab	12	-18.091	19.644	57.379	75.47	4
48	Bti	selection	12	-84.125	-16.384	51.357	135.482	4
49	Bti	total	13	0.0217	0.0228	0.0239	0.0022	4
50	Bti	shared	13	-3.959	7.181	18.321	22.28	4
51	Bti	lab	13	7.722	18.458	29.194	21.472	4
52	Bti	selection	13	-29.486	-11.277	6.933	36.419	4
53	Bti	total	14	0.0215	0.0226	0.0236	0.0021	4
54	Bti	shared	14	-0.998	9.581	20.16	21.158	4
55	Bti	lab	14	9.454	20.868	32.283	22.829	4
56	Bti	selection	14	-29.321	-11.288	6.745	36.066	4
57	Bti	total	15	0.0159	0.0169	0.0178	0.0019	4
58	Bti	shared	15	-502.029	9.807	521.643	1023.672	4
59	Bti	lab	15	-1207.532	18.043	1243.618	2451.15	4
60	Bti	selection	15	-1741.909	-8.236	1725.438	3467.347	4
61	Cop	total	1	0.0275	0.0288	0.0301	0.0026	4
62	Cop	shared	1	7.306	11.841	16.377	9.071	4
63	Cop	lab	1	13.779	17.589	21.398	7.619	4
64	Cop	selection	1	-10.937	-5.747	-0.557	10.38	4
65	Cop	total	2	0.032	0.0333	0.0347	0.0027	4
66	Cop	shared	2	12.548	15.866	19.184	6.636	4
67	Cop	lab	2	12.577	15.375	18.173	5.596	4
68	Cop	selection	2	-2.73	0.492	3.713	6.443	4

69	Cop	total	3	0.0261	0.0273	0.0285	0.0024	4
70	Cop	shared	3	2.629	7.509	12.388	9.759	4
71	Cop	lab	3	12.339	16.06	19.781	7.442	4
72	Cop	selection	3	-13.67	-8.551	-3.432	10.238	4
73	Cop	total	4	0.0339	0.0353	0.0367	0.0028	4
74	Cop	shared	4	18.714	21.813	24.913	6.199	4
75	Cop	lab	4	14.077	16.484	18.892	4.815	4
76	Cop	selection	4	2.214	5.329	8.444	6.23	4
77	Cop	total	5	0.0281	0.0293	0.0305	0.0024	4
78	Cop	shared	5	7.036	11.032	15.028	7.992	4
79	Cop	lab	5	14.15	17.245	20.34	6.19	4
80	Cop	selection	5	-10.655	-6.213	-1.772	8.883	4
81	Cop	total	6	0.0325	0.0338	0.0352	0.0027	4
82	Cop	shared	6	11.077	14.155	17.232	6.155	4
83	Cop	lab	6	12.678	14.953	17.228	4.55	4
84	Cop	selection	6	-3.749	-0.798	2.153	5.902	4
85	Cop	total	7	0.0299	0.0312	0.0325	0.0026	4
86	Cop	shared	7	21.421	25.238	29.055	7.634	4
87	Cop	lab	7	15.456	18.931	22.405	6.949	4
88	Cop	selection	7	1.969	6.307	10.645	8.676	4
89	Cop	total	8	0.0241	0.0252	0.0264	0.0023	4
90	Cop	shared	8	10.192	15.658	21.123	10.931	4
91	Cop	lab	8	14.168	19.357	24.546	10.378	4
92	Cop	selection	8	-11.233	-3.699	3.835	15.068	4
93	Cop	total	9	0.0285	0.0297	0.031	0.0025	4
94	Cop	shared	9	11.639	15.39	19.142	7.503	4
95	Cop	lab	9	13.417	16.608	19.799	6.382	4
96	Cop	selection	9	-5.41	-1.217	2.975	8.385	4
97	Cop	total	10	0.0304	0.0317	0.033	0.0026	4
98	Cop	shared	10	21.447	24.86	28.274	6.827	4
99	Cop	lab	10	15.332	18.15	20.968	5.636	4
100	Cop	selection	10	3.133	6.711	10.288	7.155	4
101	Cop	total	11	0.0281	0.0293	0.0306	0.0025	4
102	Cop	shared	11	6.433	10.954	15.475	9.042	4
103	Cop	lab	11	12.436	16.116	19.796	7.36	4
104	Cop	selection	11	-10.335	-5.162	0.011	10.346	4
105	Cop	total	12	0.0223	0.0234	0.0244	0.0021	4
106	Cop	shared	12	-3.099	4.864	12.827	15.926	4

MOLECULAR ECOLOGY

107	Cop	lab	12	9.757	15.854	21.95	12.193	4
108	Cop	selection	12	-21.689	-10.99	-0.291	21.398	4
109	Cop	total	13	0.0267	0.0279	0.0291	0.0024	4
110	Cop	shared	13	2.21	6.801	11.392	9.182	4
111	Cop	lab	13	10.492	13.943	17.395	6.903	4
112	Cop	selection	13	-12.264	-7.142	-2.021	10.243	4
113	Cop	total	14	0.0286	0.0299	0.0311	0.0025	4
114	Cop	shared	14	7.247	11.285	15.323	8.076	4
115	Cop	lab	14	12.608	15.539	18.471	5.863	4
116	Cop	selection	14	-8.636	-4.254	0.128	8.764	4
117	Cop	total	15	0.0246	0.0258	0.0269	0.0023	4
118	Cop	shared	15	8.409	14.136	19.862	11.453	4
119	Cop	lab	15	13.106	18.133	23.16	10.054	4
120	Cop	selection	15	-11.576	-3.997	3.582	15.158	4
121	Bti	total	1	0.0179	0.0189	0.0198	0.0019	5
122	Bti	shared	1	-8.773	14.51	37.794	46.567	5
123	Bti	lab	1	3.593	24.236	44.879	41.286	5
124	Bti	selection	1	-48.684	-9.726	29.233	77.917	5
125	Bti	total	2	0.0177	0.0186	0.0196	0.0019	5
126	Bti	shared	2	-53.939	6.276	66.491	120.43	5
127	Bti	lab	2	-11.454	17.763	46.98	58.434	5
128	Bti	selection	2	-95.967	-11.487	72.993	168.96	5
129	Bti	total	3	0.0201	0.0211	0.0221	0.002	5
130	Bti	shared	3	-7.507	8.14	23.786	31.293	5
131	Bti	lab	3	9.2	17.686	26.171	16.971	5
132	Bti	selection	3	-28.138	-9.546	9.047	37.185	5
133	Bti	total	4	0.02	0.0209	0.0219	0.0019	5
134	Bti	shared	4	-7.027	8.961	24.95	31.977	5
135	Bti	lab	4	10.241	18.768	27.295	17.054	5
136	Bti	selection	4	-28.699	-9.807	9.084	37.783	5
137	Bti	total	5	0.0155	0.0164	0.0172	0.0017	5
138	Bti	shared	5	-15437.727	13.629	15464.984	30902.711	5
139	Bti	lab	5	-17149.623	19.281	17188.185	34337.808	5
140	Bti	selection	5	-32608.194	-5.652	32596.889	65205.083	5
141	Bti	total	6	0.0194	0.0204	0.0214	0.002	5
142	Bti	shared	6	-8.315	8.226	24.767	33.082	5
143	Bti	lab	6	8.889	17.913	26.937	18.048	5
144	Bti	selection	6	-30.387	-9.687	11.012	41.399	5

MOLECULAR ECOLOGY

145	Cop	total	1	0.0303	0.0316	0.0329	0.0026	5
146	Cop	shared	1	14.18	17.401	20.621	6.441	5
147	Cop	lab	1	12.669	15.178	17.688	5.019	5
148	Cop	selection	1	-0.637	2.222	5.081	5.718	5
149	Cop	total	2	0.0257	0.0268	0.028	0.0023	5
150	Cop	shared	2	5.851	10.283	14.715	8.864	5
151	Cop	lab	2	12.692	15.836	18.98	6.288	5
152	Cop	selection	2	-9.348	-5.553	-1.758	7.59	5
153	Cop	total	3	0.0292	0.0304	0.0316	0.0024	5
154	Cop	shared	3	9.01	12.229	15.448	6.438	5
155	Cop	lab	3	11.647	14.136	16.625	4.978	5
156	Cop	selection	3	-4.534	-1.907	0.721	5.255	5
157	Cop	total	4	0.0308	0.032	0.0332	0.0024	5
158	Cop	shared	4	13.873	16.859	19.845	5.972	5
159	Cop	lab	4	12.608	14.72	16.832	4.224	5
160	Cop	selection	4	-0.393	2.139	4.671	5.064	5
161	Cop	total	5	0.0276	0.0287	0.0299	0.0023	5
162	Cop	shared	5	15.823	19.443	23.063	7.24	5
163	Cop	lab	5	13.728	16.48	19.232	5.504	5
164	Cop	selection	5	-0.383	2.963	6.309	6.692	5
165	Cop	total	6	0.0261	0.0272	0.0284	0.0023	5
166	Cop	shared	6	5.388	9.734	14.08	8.692	5
167	Cop	lab	6	11.53	14.594	17.658	6.128	5
168	Cop	selection	6	-8.62	-4.86	-1.1	7.52	5
169	Bti	total	1	0.0185	0.0194	0.0203	0.0018	6
170	Bti	shared	1	-14.464	9.809	34.082	48.546	6
171	Bti	lab	1	-1.771	15.415	32.6	34.371	6
172	Bti	selection	1	-16.954	-5.606	5.743	22.697	6
173	Cop	total	1	0.0283	0.0295	0.0306	0.0023	6
174	Cop	shared	1	11.283	14.484	17.686	6.403	6
175	Cop	lab	1	8.186	11.153	14.119	5.933	6
176	Cop	selection	1	0.835	3.332	5.829	4.994	6

MOLECULAR ECOLOGY

Table S2

Table S2 Results of Gene Ontology (GO) enrichment analysis for loci showing significant allele frequency changes after chronic exposure across treatments. The table lists the 45 enriched biological process GO terms identified in Bti-treated, copper-exposed, and control populations, including those shared across treatments. Terms were detected across replicate combinations showing statistically significant shared responses, with the corresponding number of replicates indicated.

Sort	GO ID	GO term	treatment	based on … replicates
1	GO:0016358	dendrite development	all	6
2	GO:0031114	regulation of microtubule depolymerization	all	5
3	GO:0007097	nuclear migration	all	4
4	GO:0006196	AMP catabolic process	all	4
5	GO:0042981	regulation of apoptotic process	Bti	6
6	GO:0006952	defense response	Bti	5
7	GO:0000122	negative regulation of transcription by RNA polymerase II	Bti	5
8	GO:0045893	positive regulation of DNA-templated transcription	Bti	4
9	GO:0006338	chromatin remodeling	Bti	4
10	GO:0006741	NADP biosynthetic process	Bti	4
11	GO:0019674	NAD metabolic process	Bti	4
12	GO:0006997	nucleus organization	Bti	4
13	GO:0006357	regulation of transcription by RNA polymerase II	Bti	4
14	GO:0033499	galactose catabolic process via UDP-galactose	Bti	4
15	GO:0000184	nuclear-transcribed mRNA catabolic process, nonsense-mediated decay	Bti	4
16	GO:0007602	phototransduction	Copper	5
17	GO:0046513	ceramide biosynthetic process	Copper	5
18	GO:0006606	protein import into nucleus	Copper	5
19	GO:0043171	peptide catabolic process	Copper	5
20	GO:0000012	single strand break repair	Copper	4
21	GO:0006535	cysteine biosynthetic process from serine	Copper	4
22	GO:1902275	regulation of chromatin organization	Copper	4
23	GO:0008543	fibroblast growth factor receptor signaling pathway	Copper	4
24	GO:0007190	activation of adenylate cyclase activity	Copper	4
25	GO:0019343	cysteine biosynthetic process via cystathionine	Copper	4
26	GO:0006303	double-strand break repair via nonhomologous end joining	Copper	4
27	GO:0061512	protein localization to cilium	Copper	4
28	GO:0051295	establishment of meiotic spindle localization	Control	5
29	GO:0061077	chaperone-mediated protein folding	Control	5
30	GO:0006487	protein N-linked glycosylation	Control	5
31	GO:0007051	spindle organization	Control	5
32	GO:0044878	mitotic cytokinesis checkpoint signaling	Control	4

MOLECULAR ECOLOGY

33	GO:0046901	tetrahydrofolylpolyglutamate biosynthetic process	Control	4
34	GO:0009838	abscission	Control	4
35	GO:0032979	protein insertion into mitochondrial inner membrane from matrix	Control	4
36	GO:0070085	glycosylation	Control	4
37	GO:0006325	chromatin organization	Control	4
38	GO:0007156	homophilic cell adhesion via plasma membrane adhesion molecules	Control-Bti	5
39	GO:0045104	intermediate filament cytoskeleton organization	Control-Bti	4
40	GO:0042060	wound healing	Control-Bti	4
41	GO:0031122	cytoplasmic microtubule organization	Control-Bti	4
42	GO:0000492	box C/D snoRNP assembly	Control-Bti	4
43	GO:0034244	negative regulation of transcription elongation by RNA polymerase II	Control-Copper	5
44	GO:0006685	sphingomyelin catabolic process	Control-Copper	5
45	GO:0019919	peptidyl-arginine methylation, to asymmetrical-dimethyl arginine	Control-Copper	4

MOLECULAR ECOLOGY

Table S3

Table S3 Results of Pearson correlation analyses between genomic differentiation (PC1 and PC2 scores) and phenotypic endpoints in C. riparius *populations from the Bti-selection experiment exposed to 33% FR Bti during a later larval stage on day 10 of the sensitivity test (Kolbenschlag et al., 2024). The table reports the Pearson correlation p-value, the Benjamini–Hochberg false discovery rate (BH-FDR)–adjusted p-value, and the coefficient of determination (R^2). A single statistically significant association was detected between PC2 and female protein content (marked with "**"). However, the corresponding plot (Figure S9) shows that this pattern is unrelated to exposure history.*

**Predictor**	**Response**	**P value**	**BH FDR**	**R^2**
PC1 score	Total emergence	0.223	0.579	0.279
PC2 score	Total emergence	0.784	0.937	0.016
PC1 score	Female emergence	0.278	0.579	0.229
PC2 score	Female emergence	0.237	0.579	0.265
PC1 score	Male emergence	0.810	0.937	0.013
PC2 score	Male emergence	0.571	0.837	0.069
PC1 score	Time to 50% emergence (f)	0.514	0.808	0.090
PC2 score	Time to 50% emergence (f)	0.342	0.579	0.181
PC1 score	Time to 50% emergence (m)	0.886	0.953	0.005
PC2 score	Time to 50% emergence (m)	0.312	0.579	0.202
PC1 score	Female weight	0.749	0.937	0.022
PC2 score	Female weight	0.114	0.501	0.423
PC1 score	Male weight	0.937	0.953	0.001
PC2 score	Male weight	0.062	0.447	0.533
PC1 score	Lipid content females	0.798	0.937	0.014
PC2 score	Lipid content females	0.317	0.579	0.198
PC1 score	Lipid content males	0.325	0.579	0.192
PC2 score	Lipid content males	0.953	0.953	0.001
PC1 score	Protein content females	0.142	0.521	0.378
PC2 score	Protein content females	0.002 **	0.040 **	0.879
PC1 score	Protein content males	0.073	0.447	0.505
PC2 score	Protein content males	0.081	0.447	0.487

MOLECULAR ECOLOGY

Table S4

Table S4 Results of Pearson correlation analyses between genomic differentiation (PC1 and PC2 scores) and phenotypic endpoints in C. riparius *populations from the copper-selection experiment exposed to 100 mg/kg dw Cu (Pietz et al., 2025). The table reports the Pearson correlation p-value, the Benjamini–Hochberg false discovery rate (BH-FDR)–adjusted p-value, and the coefficient of determination (R^2). No consistent or robust associations were detected.*

**Predictor**	**Response**	**P value**	**BH FDR**	**R^2**
PC1 score	Total emergence	0.537	0.990	0.039
PC2 score	Total emergence	0.339	0.990	0.092
PC1 score	Female emergence	0.597	0.990	0.029
PC2 score	Female emergence	0.519	0.990	0.043
PC1 score	Male emergence	0.750	0.990	0.011
PC2 score	Male emergence	0.472	0.990	0.053
PC1 score	Time to 50% emergence (f)	0.513	0.990	0.044
PC2 score	Time to 50% emergence (f)	0.854	0.990	0.004
PC1 score	Time to 50% emergence (m)	0.941	0.990	0.001
PC2 score	Time to 50% emergence (m)	0.810	0.990	0.006
PC1 score	Female weight	0.198	0.990	0.160
PC2 score	Female weight	0.168	0.990	0.181
PC1 score	Male weight	0.899	0.990	0.002
PC2 score	Male weight	0.582	0.990	0.031
PC1 score	ALA levels female	0.313	0.990	0.102
PC2 score	ALA levels female	0.317	0.990	0.100
PC1 score	ALA levels male	0.874	0.990	0.003
PC2 score	ALA levels male	0.133	0.990	0.211
PC1 score	ARA levels female	0.882	0.990	0.002
PC2 score	ARA levels female	0.933	0.990	0.001
PC1 score	ARA levels male	0.608	0.990	0.027
PC2 score	ARA levels male	0.598	0.990	0.029
PC1 score	Omega-3 females	0.212	0.990	0.151
PC2 score	Omega-3 females	0.992	0.992	0.000
PC1 score	Omega-3 males	0.689	0.990	0.017
PC2 score	Omega-3 males	0.334	0.990	0.093
PC1 score	Omega-6 females	0.957	0.990	0.000
PC2 score	Omega-6 females	0.686	0.990	0.017
PC1 score	Omega-6 males	0.696	0.990	0.016
PC2 score	Omega-6 males	0.836	0.990	0.005
